# Real-time monitoring of circadian clock oscillations in primary cultures of mammalian cells using Tol2 transposon-mediated gene transfer strategy

**DOI:** 10.1186/1472-6750-10-3

**Published:** 2010-01-22

**Authors:** Kazuhiro Yagita, Iori Yamanaka, Noriaki Emoto, Koichi Kawakami, Shoichi Shimada

**Affiliations:** 1 Department of Neuroscience and Cell Biology, Osaka University Graduate School of Medicine, Yamadaoka 2-2, Suita, Osaka, 565-0871 Japan; 2 COE Unit of Circadian Systems, Nagoya University Graduate School of Science, Furo-cho, Chikusa-ku, Nagoya 464-8602, Japan; 3 Department of Cardiovascular Medicine, Kobe University Graduate School of Medicine, Kusunoki-cho, Chuo-ku, Kobe, Japan; 4 Department of Molecular and Developmental Biology, National Institute of Genetics, The Graduate University for Advanced Studies (SOKENDAI), 1111 Yata, Mishima, Shizuoka 411-8540, Japan; 5 Department of Genetics, The Graduate University for Advanced Studies (SOKENDAI), 1111 Yata, Mishima, Shizuoka 411-8540, Japan; 6 Group of Brain Function and Structure, Nagoya University Graduate School of Science, Furo-cho, Chikusa-ku, Nagoya 464-8602, Japan

## Abstract

**Background:**

The circadian rhythm in mammals is orchestrated by a central pacemaker in the brain, but most peripheral tissues contain their own intrinsic circadian oscillators. The circadian rhythm is a fundamental biological system in mammals involved in the regulation of various physiological functions such as behavior, cardiovascular functions and energy metabolism. Thus, it is important to understand the correlation between circadian oscillator and physiological functions in peripheral tissues. However, it is still difficult to investigate the molecular oscillator in primary culture cells.

**Results:**

In this study, we used a novel Tol2 transposon based *Dbp *promoter or *Bmal1 *promoter driven luciferase reporter vector system to detect and analyze the intrinsic molecular oscillator in primary culture cells (mouse embryonic fibroblasts, fetal bovine heart endothelial cells and rat astrocytes). The results showed circadian molecular oscillations in all examined primary culture cells. Moreover, the phase relationship between *Dbp *promoter driven and *Bmal1 *promoter driven molecular rhythms were almost anti-phase, which suggested that these reporters appropriately read-out the intrinsic cellular circadian clock.

**Conclusions:**

Our results indicate that gene transfer strategy using the Tol2 transposon system of a useful and safe non-viral vector is a powerful tool for investigating circadian rhythms in peripheral tissues.

## Backgrounds

The circadian clock is driven by a stable and robust self-sustaining molecular oscillator. This oscillation machinery resides in most of the cells in our body, and even cultured cell lines also have an intrinsic circadian oscillator [1-3]. The molecular oscillation of circadian clock consists of interlocked positive and negative transcription/translation feedback loops (TTFL) involving a set of clock genes, and clock-controlled output genes that link the oscillator to clock-controlled processes [4]. CLOCK and BMAL1 are basic-helix-loop-helix (bHLH) PAS transcription factors that heterodimerize and transactivate the core clock components such as *Period *(*Per1,2,3*), *Cryptochrome *(*Cry1 *and *Cry2*) and *Rev-Erbα *[4-6]. Then, PER and CRY proteins suppress the activity of the CLOCK/BMAL1, whereas REV-ERB α suppresses *Bmal1 *gene expression.

Promoters of clock genes that show cyclic transcription often contain circadian enhancers such as E-box and RRE (Ror responsive element), and these circadian enhancer-containing promoters can drive cyclic expression of luciferase gene as a reporter [7]. Using the bioluminescence reporters, we recently established real-time circadian clock analysis system in living cells [8].

It is important to investigate the correlation between circadian oscillator and physiological functions in each differentiated cell. However, it is difficult to investigate the molecular oscillator of primary culture cells because of the difficulty to read-out the intrinsic circadian rhythm. In this study, we describe the successful detection and analysis of the intrinsic molecular oscillator in primary culture cells such as mouse embryonic fibroblasts (MEF), fetal bovine heart endothelial cells (FBHE) and rat astrocytes using Tol2 transposon-based vector system. The Tol2 transposon system is a useful method to generate stably transfected luciferase-expressing cells even in primary culture cells. We were able to monitor circadian clock oscillations by real-time monitor system. We propose that the Tol2 transposon-based vector system is a simple and safe non-viral technique that can be used for various types of cells.

## Results

### Efficiency of Tol2 transposon-based gene transfer

**Figure 1:**
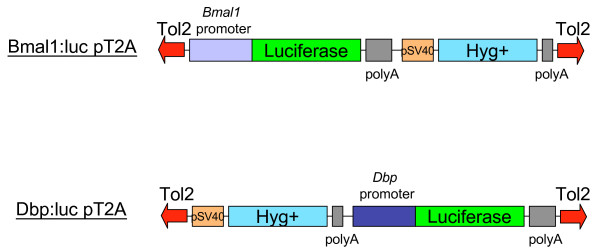
**Tol2 based luciferase reporter constructs to read-out circadian molecular oscillation in mammalian cells**. Bmal1:luc-pT2A construct contains 0.5 kb-long mouse *Bmal1 *promoter driven luciferase cDNA and hygromycin B-resistant cassette (Hyg+). The Dbp:luc-pT2A construct contains hygromycin B-resistant cassette (Hyg+) and 0.6 kb-long mouse *Dbp *promoter driven luciferase cDNA (see Methods).

**Figure 2:**
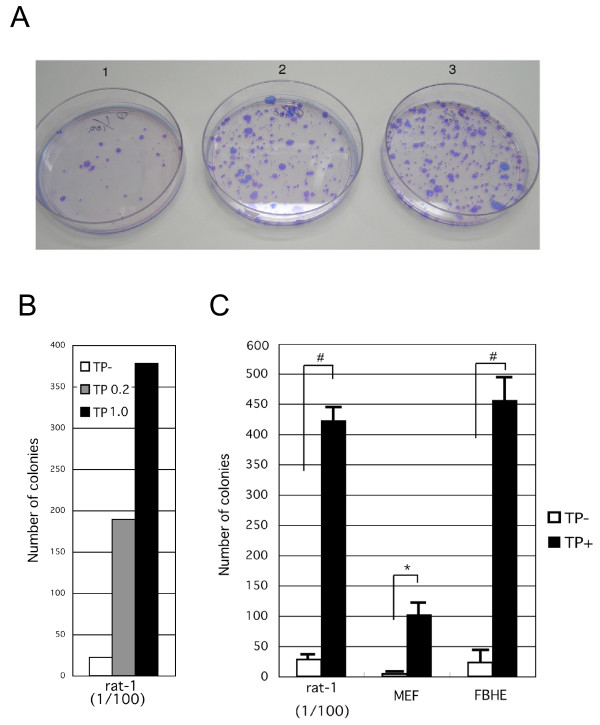
**Tol2 transposon-based vector system can generate stably transfected cells with extremely high efficiency**. (A) Colony formation assay examining the efficiency generating Dbp:luc-pT2A stably transfected rat-1 cells. Dish 1: negative control condition without co-transfection of transposase expression plasmid (pCAGGS-TP). Dish 2: transfection condition of 1 μg of Dbp:luc-pT2A plasmid and 0.2 μg of pCAGGS-TP plasmid. Dish 3: transfection condition of 1 μg of Dbp:luc-pT2A plasmid and 1 μg of pCAGGS-TP plasmid. The cells were fixed and stained after two weeks selection culture. (B) Quantitative data of the dishes shown in (A). (C) Tol2 transposon vector system allows the generation of Dbp:luc-pT2A stably transfected cells with over 20-fold higher efficiency compared with the system lacking co-transfection of pGAGGS-TP plasmid. Data are mean ± SEM numbers of cell colonies. Hash indicates P < 0.005, Asterisk indicates P < 0.05, t-test (n = 3)

We generated the Tol2 based reporter expression constructs, *mBmal1 *promoter: luciferase (Bmal1:luc) and *mDbp *promoter: luciferase (Dbp:luc) containing hygromycin-resistant gene as a selection marker (Fig. 1). It is known that the *Bmal1 *and *Dbp *promoters exhibit circadian transcriptional activity rhythms, because they contain RRE and E-box enhancer elements, respectively [9]. First, using these Tol2-based circadian reporter constructs, colony formation assay was performed to evaluate the efficiency of Tol2-mediated reporter gene insertion into the genome of cultured mammalian cells (Fig. 2A). Dbp:luc-pT2A plasmid was transfected into rat-1 fibroblast cell line with or without Tol2 transposase expression plasmid (pCAGGS-TP). As shown in Fig. 2A and 2B, co-expression of Tol2 transposase markedly increased hygromycin-resistant Dbp:luc-pT2A stably transformed colonies (Fig. 2A and 2B). Quantitative analysis indicated that co-expression of transposase increased the efficiency of the formation of Dbp:luc stably transformed rat-1 cells to over 17-fold, compared with transposase non-expressing condition (Fig. 2B).

Since these results suggested that Tol2 transposon-based vector system markedly improved transgenic efficiency in cultured mammalian cell line, we next examined whether Tol2 transposon was effective also in stable transfection into primary cultures of MEF, rat astrocytes and FBHE cells. Among these, rat astrocytes were difficult to evaluate the efficiency of stable transfection, because they did not form colonies and the viability rate after transfection was too low for meaningful quantitative analysis. In this assay, Bmal1:luc-pT2A plasmid was used to exclude possible plasmid-specific events. Quantitative analysis showed extremely high efficient stable transfection rates in not only rat-1 cell line but also both MEF and FBHE cells (Fig. 2C). In addition to Dbp:luc-pT2A vector, the Bmal1:luc-pT2A vector also allowed the generation of stably transfected rat-1 cell line with over 18-fold higher efficiency, under Tol2 transposase expression. Strikingly, the expression of transposase allowed the generation of both MEF and FBHE cells stably transfected with Bmal1:luc-pT2A with extremely higher efficiency (24-fold and 21-fold, respectively) (Fig. 2C). These results also indicate that our Tol2 transposon system is an useful tool for gene transfer into mammalian primary culture cells.

### Real-time monitor of circadian clock oscillation in Dbp:luc or Bmal1:luc stably transfected primary culture cells

**Figure 3:**
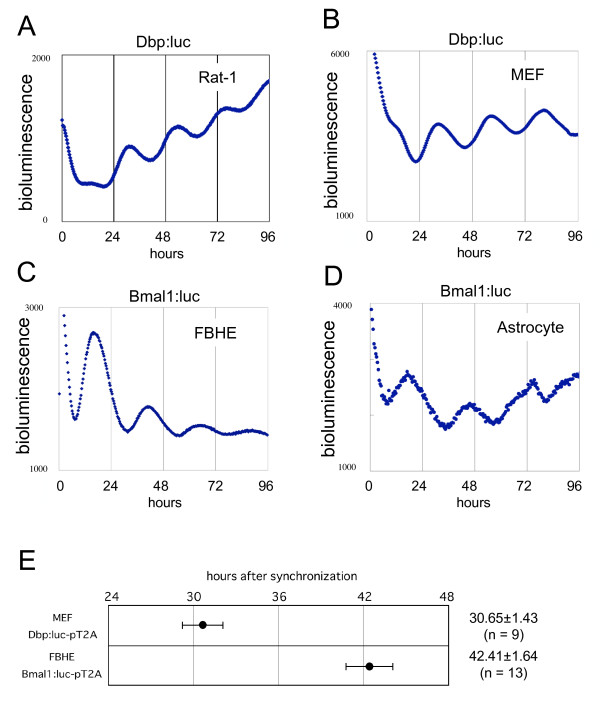
**Real-time monitoring of circadian molecular oscillations by PMT in rat-1, MEF, FBHE and rat astrocytes**. Photomultiplier-tube (PMT)-based bioluminescence was monitored in Dbp:luc-pT2A and Bmal1:luc-pT2A stably transfected cells. (A) Rat-1 cells stably transfected with Dbp:luc-pT2A showing circadian bioluminescence oscillations. A representative example of 12 samples with similar results. (B) MEFs stably transfected with Dbp:luc-pT2A showing circadian bioluminescence oscillations. A representative example of 9 samples with similar results. Note the similar phases of circadian oscillations in rat-1 and MEFs, although the cycle duration is slightly different in the two cells. (C) Fetal bovine heart endothelial (FBHE) cells stably transfected with Bmal1:luc-pT2A showing circadian bioluminescence oscillations. A representative example of 13 samples with similar results. (D) Rat astrocytes stably transfected with Bmal1:luc-pT2A showing circadian bioluminescence oscillations. A representative example of 4 samples with similar results. (E) Bmal1:luc-pT2A driven bioluminescence in FBHE cells exhibits almost anti-phasic rhythms relative to the Dbp:luc-pT2A driven bioluminescence rhythms shown in MEF cells. Peak phases of Dbp:luc-pT2A stably transfected MEFs (n = 9) and Bmal1:luc-pT2A stably transfected FBHE cells (n = 13). Mean phase ± SD is indicated to the right of the figure.

Dbp:luc-pT2A and Bmal1:luc-pT2A plasmids were generated to analyze the cellular circadian clock oscillation in various types of cultured cells. Both Dbp:luc and Bmal1:luc are available for circadian clock analysis in rat-1 fibroblast cell line [9]. In this study, using our Tol2 transposon system, we obtained Dbp:luc or Bmal1:luc reporter-stably expressing MEFs, rat astrocytes and FBHE cells. Real-time monitoring of luciferase activity was performed to investigate whether the reporter transgene using Tol2 transposon is suitable for circadian clock analysis in cultured mammalian cells. Fig. 3 shows circadian oscillation of Dbp:luc and Bmal1:luc driven bioluminescence in all types of cells including primary culture cells (Fig. 3A, B, C and 3D). In these studies, rat-1 and MEF were transfected with Dbp:luc-pT2A, while rat astrocytes and FBHE cells were transfected with Bmal1:luc-pT2A plasmids, with transposase expression plasmid (Fig. 3). All bioluminescence positive lines of Bmal1:luc-pT2A stably transfected or Dbp:luc-pT2A stably transfected cells showed circadian oscillation of Bmal1:luc or Dbp:luc driven bioluminescence. Importantly, the oscillatory phases of Dbp:luc-pT2A and Bmal1:luc-pT2A transfected cells were almost anti-phase (Fig. 3E), suggesting that these cells correctly represented the phase relation of endogenous *Dbp *and *Bmal1 *gene expression patterns. Previous studies reported that *Dbp *and *Bmal1 *genes are expressed nearly anti-phase in various types of cells and tissues [7,10,11]. Considered together, these results indicate that the Tol2 transposon-based vector system is useful and powerful tool for investigating the circadian clock function in various functionally differentiated primary cultured cells.

## Discussion

In this study, first we showed that the Tol2 transposon system is a useful technique in generating stable transfected primary culture cells and, second, we demonstrated that the Tol2 transposon system is applicable to the study of circadian clock oscillations.

The Tol2 transposon was originally discovered from Medaka fish (Orzyias latipes) [12]. An active autonomous member of Tol2 was first identified by the analysis using zebrafish embryos [13]. Since then, the Tol2 transposon system has been mainly used for random insertion mutagenesis and transgene in zebrafish [14]. Although recent reports have indicated that the transposon systems such as piggyBac and SleepingBeauty in addition to Tol2 are also active in mammalian cells [15,16], few studies have been reported that utilized the Tol2 system for transfection to mammalian primary culture cells. In the present study, we showed that the Tol2 transposon system is a useful tool in generating stable transfected primary culture cells such as MEF and fetal bovine heart endothelial cells.

As previously reported, transfection of primary cells have been also performed by using retroviral vectors [17]. Comparing with the retroviral vectors, the number of the stably transfected cells obtained after selection culture is small in Tol2 transposon system. However, the handling is easier because Tol2 transposon system requires only co-transfection of two plasmids. Furthermore, it is much safer than retroviral vectors. These features should be enough reason to choose the Tol2 transposon system for many researchers instead of retroviral vector system.

We demonstrated that the Tol2 transposon system is applicable to the study of circadian clock oscillation. In this study, we were able to detect circadian clock oscillations in primary culture cells including MEF, astrocytes and FBHE cells expressing *Bmal1 *promoter- or *Dbp *promoter-driven luciferase. All generated cells exhibited robust daily cycles of bioluminescence, and there was no obvious difference of observed features between Tol2-system and usual DNA transfection methods experienced before [8,18].

In obtained stably transfected cells using Tol2 system, the oscillation phase was almost identical; even different types of cells such as FBHE cells and astrocytes were stably integrated with Bmal1:luc reporter by the Tol2 system (Fig. 3A-D). Moreover, as seen in endogenous gene expression patterns, these cells showed almost opposite phases of Bmal1:luc driven rhythms and Dbp:luc driven rhythms (Fig. 3E). These phase-relationship of circadian oscillation indicate that the integrated reporter correctly read-out the intrinsic circadian clock system.

In addition, our results are the first to demonstrate circadian clock oscillations in primary endothelial cells in real-time manner. Previous studies suggested the importance of circadian regulation of endothelial function for various kinds of physiological and pathological events such as blood pressure and endothelial proliferation [19]. However, little is known about the molecular relationship between circadian clock and endothelial function, because it has been difficult to analyze the circadian oscillation of primary endothelial cells.

Viral vector system is also very high-efficient gene transfer tools. Getting together, Tol2 transposon based vector system is useful and safe tool to investigate the circadian clock in various types of primary culture cells.

## Conclusions

The Tol2 transposon-based assay system described in this study should enhance the biological analysis of molecular oscillator of circadian clock and the function of primary culture cells such as MEFs, rat astrocytes and fetal bovine heart endothelial cells. We were able to monitor the circadian clock oscillations by real-time monitor system in these cells. We propose that the Tol2 transposon-based vector system is a simple and safe non-viral technique that can be used for various types of cells.

## Methods

### Plasmids

For Bmal1:luc-pT2A vector, 0.5 kb of the 5' flank region of mouse *Bmal1 *gene was cloned by PCR into pCR2.1-Topo vector. The Bmal1 promoter fragment was digested with *Bgl*II and *Hind*III, then subcloned into *Bgl*II/*Hind*III site of pGL4.15 vector [8]. The *Bgl*II/*Sal*I digested fragment of Bmal1-pro-pGL4.15, containing Bmal1 promoter, Luciferase and hygromycin-resistant gene, was subcloned into the *Bgl*II/*Xho*I site of the pT2AL200R150G vector.

For Dbp:luc-pT2A vector, the *mDbp *promoter:luciferase reporter construct (Dbp:luc-pGL4.11) was generated as reported previously [9]. The Dbp promoter, luciferase fragment and hygromycin-resistant gene fragment obtained from Dbp:luc-pT2A and pTRE2-Hyg vectors were subcloned into the *Xho*I/*Bgl*II digested pT2AL200R150G Tol2 vector.

### Cell culture and transfection

Rat-1 and mouse embryonic fibroblasts (MEF) were cultured in Dulbecco's modified Eagle medium (DMEM) with 10% fetal bovine serum (FBS). FBHE cells were cultured in DMEM with 10% FBS and 2 ng/ml of basic fibroblast growth factor (bFGF). Rat astrocytes were obtained from Human Science Research Resources Bank (HSRRB, Osaka, Japan), and cultured in DMEM with 10% FBS.

For transfection, 1 μg of Dbp:luc-pT2A or Bmal1:luc-pT2A and 1 μg of pCAGGS-TP or pcDNA3 plasmids were transfected using Fugene 6 reagent into 3 × 10^5 ^cells cultured in a 6-well plate. After overnight culture, the cells were trypsinized and plated in 9-cm dishes with culture medium for each type of cells as indicated above. In rat-1 cells, 1/100 of transfected cells were plated in 9-cm dishes. Selection with 250 μg/ml hygromycin-containing medium was started 24 hours after plating on the 9-cm dishes. After two or three weeks, surviving cell colonies were picked up using cloning rings. For rat astrocytes, cells did not form the colonies, thus surviving cells were gathered and cultured in 6- cm dishes after selection.

### Real-time monitoring of cellular circadian oscillation

For real-time monitoring of cellular circadian oscillation, 1 × 10^5 ^hygromycin-resistant cells (rat-1, MEF, astrocytes and FBHE cells) were seeded in 24-well plates and cultured overnight with growth medium of each type of cells. Next, the medium was changed with a medium containing DMEM/F12, 10% FBS, 15 mM 4-(2-hydroxyethyl)-1-piperazineethanesulfonic acid (HEPES), 0.2 mM luciferin and 100 nM dexamethasone to synchronize the cellular circadian clocks. Bioluminescence was monitored using a photomultiplier-tube-based bioluminescence monitoring system [8]. The bioluminescence was measured for 60 sec in every 20 minutes.

### Phase analysis of cellular circadian oscillation

Phase analysis of cellular circadian rhythms are analyzed as previously reported [2,20]. Briefly, second peaks after ssynchronization of bioluminescence of Dbp:luc-pT2A stably transfected MEF lines (n = 9) and second peaks of Bmal1:luc-pT2A stably transfected FBHE lines (n = 13) are determined using detrended and smoothed PMT based bioluminescence data.

## Authors' contributions

KY designed and performed experiments, analyzed data and wrote the paper. IY and NE performed experiments. KK provided the Tol2 transposon basic vectors. SS assisted performing experiments.
